# Eruptive Lichen Planus Associated With Chronic Hepatitis C Infection Presenting as a Diffuse, Pruritic Rash

**DOI:** 10.7759/cureus.9732

**Published:** 2020-08-14

**Authors:** Paige B Beck, Mustafa Goksel, Shashank Kraleti

**Affiliations:** 1 Department of Family and Preventive Medicine, University of Arkansas for Medical Sciences, Little Rock, USA; 2 Department of Pathology, University of Arkansas for Medical Sciences, Little Rock, USA

**Keywords:** hepatitis c, diffuse rash, eruptive, lichen planus

## Abstract

Lichen planus has been associated with several precipitating factors, such as drugs, immunizations, and viral infections, including hepatitis C virus (HCV). Eruptive or disseminated lichen planus is a rare variation that most often presents as an acute, widespread exanthem that progresses rapidly and usually lasts for a shorter duration. This variation has not been well studied, and little is known about the etiologies and treatments of this rare form. Thus far, only a few cases of eruptive lichen planus have been reported to be associated with HCV infection. We report a case a 62-year-old woman who presented with a rapidly progressive, diffuse, pruritic rash of the trunk, upper extremities, and thighs that was determined to be eruptive lichen planus secondary to chronic HCV infection. The patient was treated with topical steroids and oral antihistamines, and her rash spontaneously resolved approximately six months after the initial presentation.

## Introduction

Lichen planus is a mucocutaneous disease that usually presents insidiously and tends to follow the course of a typical chronic disease. The classic presentation of cutaneous lichen planus is characterized by the development of pruritic, flat-topped, violaceous, polygonal papules distributed mainly over the wrists, arms, and legs. The papules often coalesce into plaques, which may have fine, whitish lines (known as Wickham’s striae) over the surface and can be associated with hyperpigmentation after resolution [1]. 

The prevalence of cutaneous lichen planus is estimated to be at less than 1% of the population, though the epidemiology of lichen planus is not well defined. It may exhibit numerous variations in pattern that include hypertrophic, atrophic, bullous, erosive, follicular, actinic, annular, pigmented, linear, and eruptive. It may involve any part of the body, including the skin, mucous membranes, genitalia, nails, and scalp [2]. Eruptive lichen planus is a rare variation that most often presents as an acute, widespread exanthem that progresses rapidly, and usually lasts for a shorter duration [1,3]. This variation has not been well studied, and little is known about the etiologies and treatments of this rare form. Thus far, only a few cases of eruptive lichen planus have been reported to be associated with hepatitis C virus (HCV) infection.

## Case presentation

**Figure 1:**
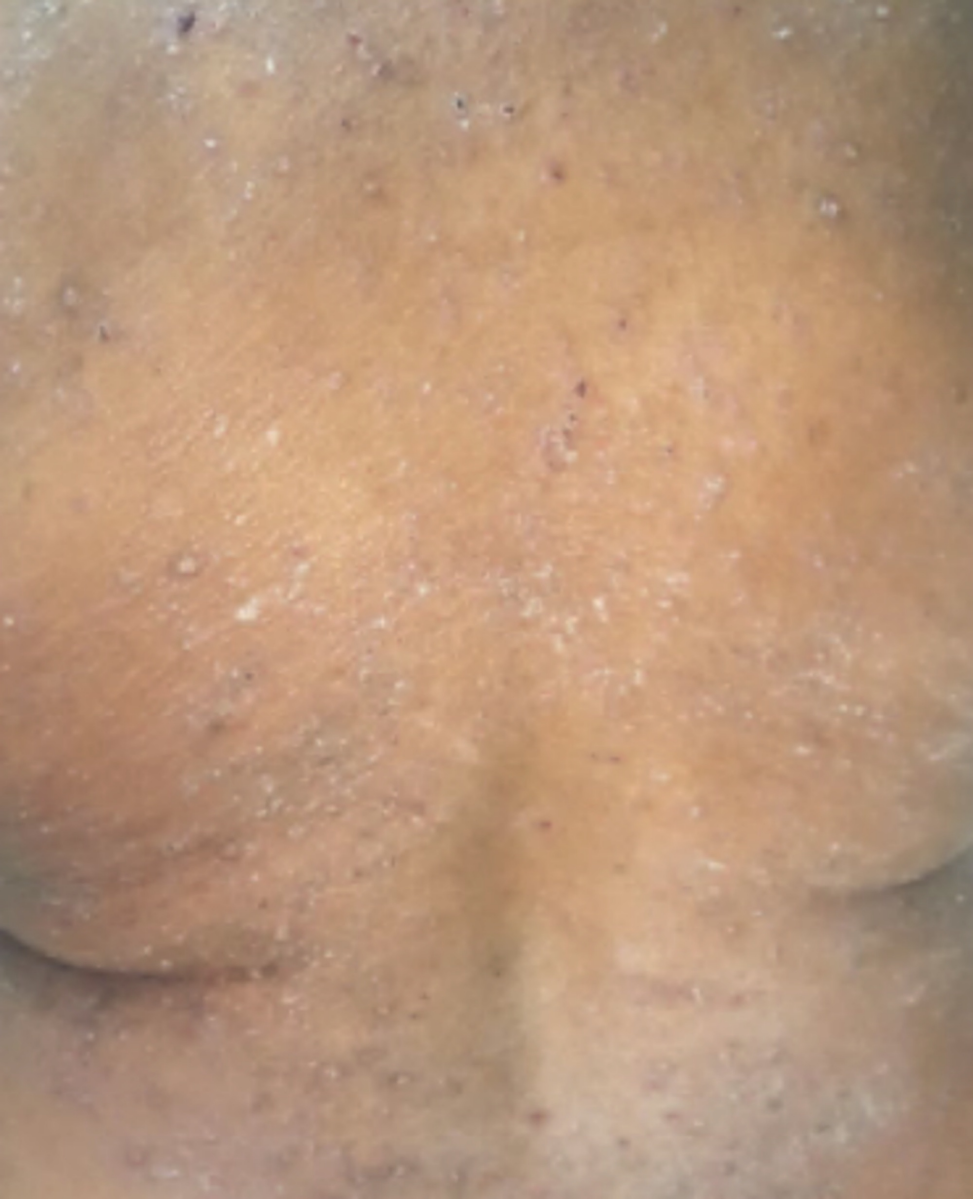
Diffuse, scaly papules with extensive excoriations overlying the trunk

A 62-year-old African American woman presented to her family physician’s office with the complaint of a pruritic rash that had been present for approximately one week. A 14-point review of systems was negative except for the rash. The patient reported no recent travel or sick contacts. At the time of presentation, her medications included aspirin, atenolol, nifedipine, and gabapentin. The patient denied any recent changes in medications except for gabapentin, which was started about two months earlier. On physical examination, the patient was afebrile and rest of the vital signs were stable. Examination of the skin showed numerous mildly erythematous, scaly papules with extensive excoriations overlying the trunk, upper extremities, and thighs (Figure 1). 

The patient’s medical history was significant for hypertension, vitamin D deficiency, untreated HCV infection, and polysubstance abuse, including alcohol and cocaine. The patient’s history of allergies included angioedema with penicillin.

**Figure 2:**
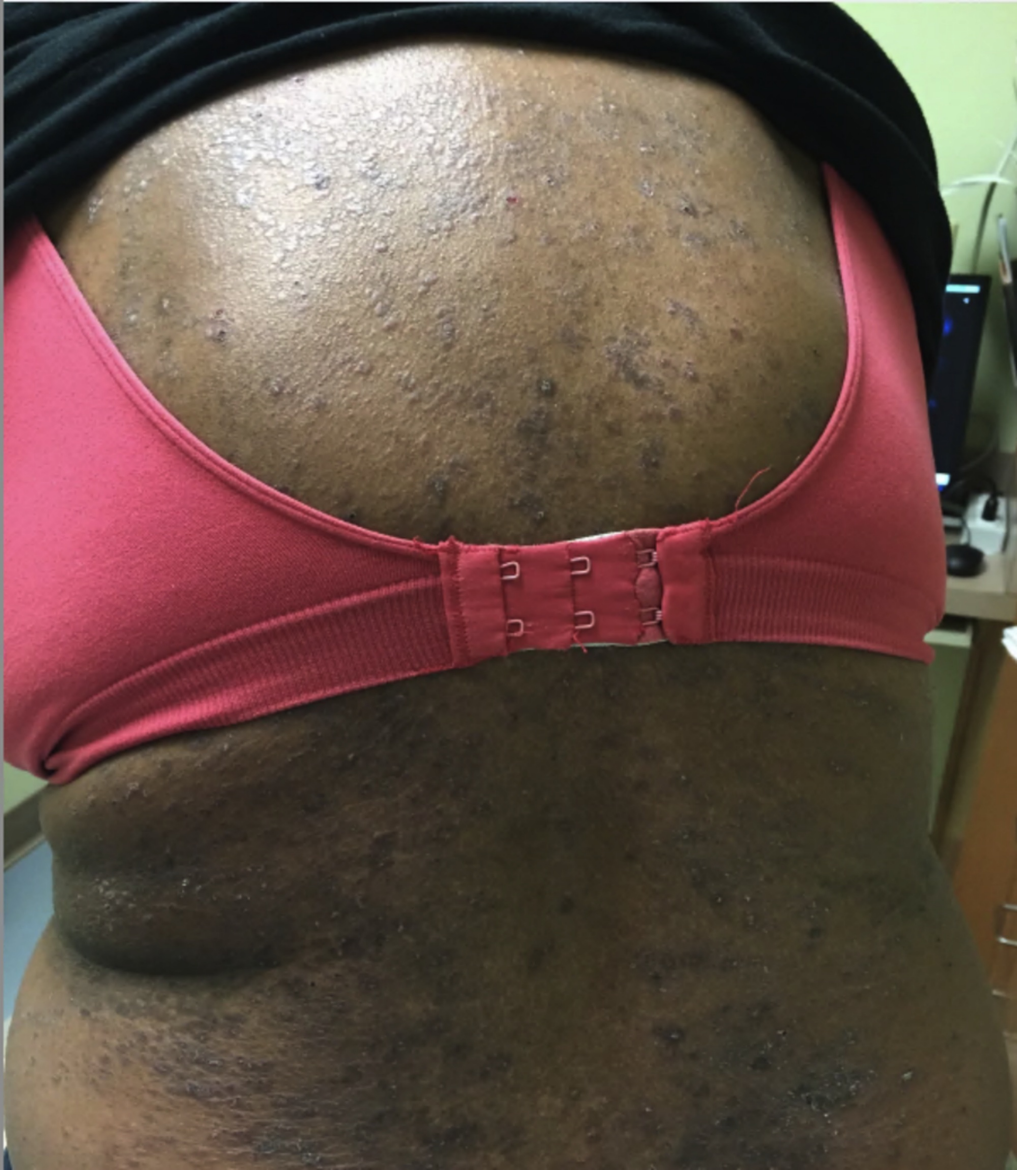
Purple flat-topped papules coalescing into plaques on the trunk, some with an overlying scale

Gabapentin was discontinued due to suspected drug reaction, and the patient was started on 1% triamcinolone cream and hydroxyzine 25 mg three times a day. Two weeks later, the patient presented to clinic with worsening rash and pruritus. The rash had progressed into purple flat-topped papules coalescing into plaques, some with an overlying scale (Figure 2).

**Figure 3:**
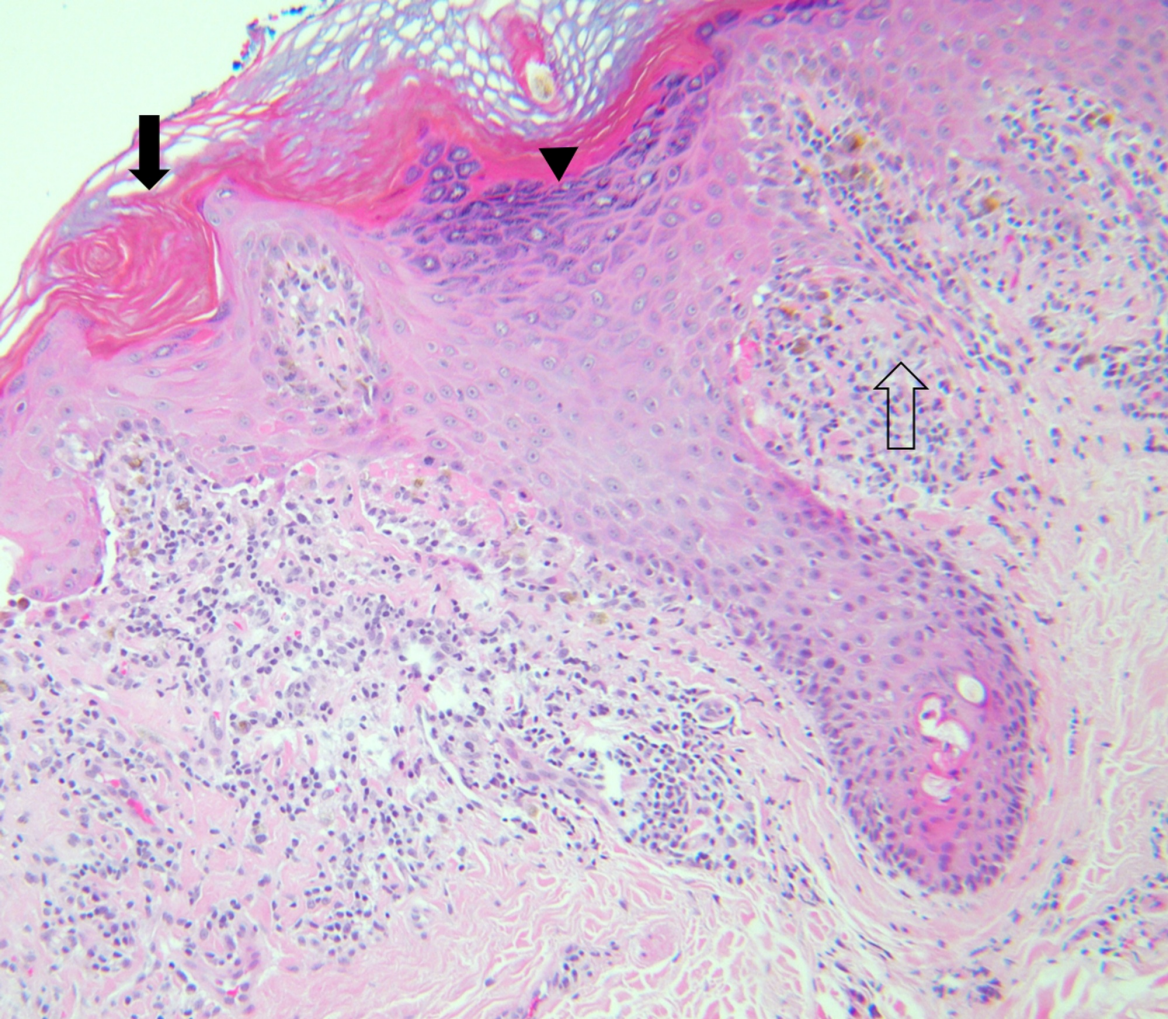
Biopsy demonstrating hyperkeratosis (closed arrow), hypergranulosis (arrowhead), and rete “saw-tooth” ridges (open arrow) (H&E, ×100)

**Figure 4:**
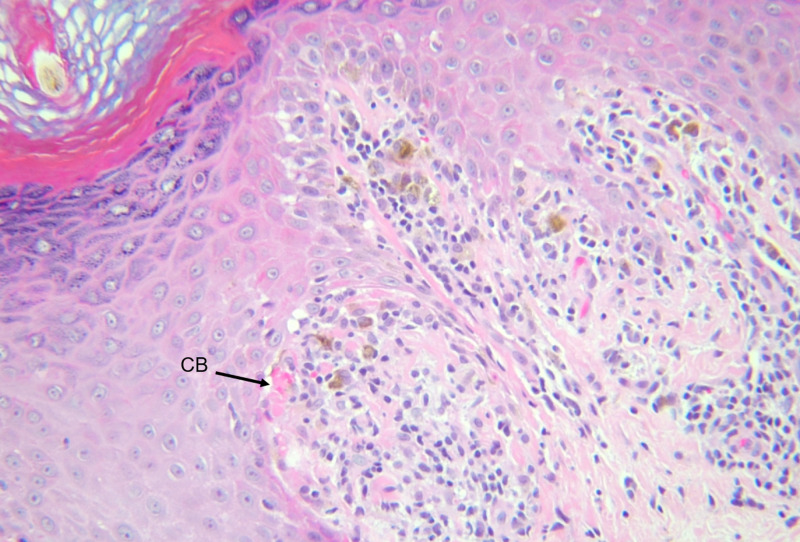
Biopsy demonstrating civatte bodies (CB) (H&E, ×200)

Three punch biopsies were performed from the patient’s upper extremity and trunk, and the patient was referred to dermatology. The pathology of all three punch biopsies was consistent with lichen planus (Figures 3, 4).

The dermatologist determined that the patient’s presentation was consistent with disseminated/eruptive lichen planus due to untreated hepatitis C infection. The patient was started on desoximetasone ointment twice a day, and hydroxyzine was continued as needed for pruritis. The patient opted to continue to consume alcohol; therefore, she was not considered a candidate for the treatment of hepatitis C infection. The rash resolved approximately six months after her initial presentation.

## Discussion

Lichen planus is mostly a clinical diagnosis, but biopsies can be obtained and sent for histology to confirm the diagnosis, especially in cases that do not present classically. Typical histopathologic features of lichen planus include hyperkeratosis, vacuolization of the basal layer, hypergranulosis, “saw-tooth” shaped rete ridges, band-like lymphocytic infiltrate at the dermal-epidermal junction, and apoptotic keratinocytes in the papillary dermis known as civatte bodies [2].

Lichen planus has been found to be associated with several precipitating factors, such as drugs, immunizations, and viral infections. There is an abundance of literature, including systemic reviews, meta-analyses, case-control studies, and case reports that have shown a significant association between lichen planus and infection with HCV [4-7]. The estimated prevalence of HCV in patients with lichen planus varies from 4% to 24% [8]. Two meta-analyses showed that patients with lichen planus were approximately five times as likely as controls to be HCV seropositive; moreover, lichen planus was 2.5 to 4.5 times more likely to develop in the HCV-seropositive patients [4,5]. Although most commonly reported variant of HCV-associated lichen planus in literature was oral lichen planus, studies have also shown that a significant percentage of patients with cutaneous lichen planus have HCV antibody. Thus far, only a few cases of eruptive lichen planus have been reported to be associated with HCV infection [9,10].

Treatment of lichen planus depends on the location and severity of the disease. Without treatment, approximately 68% of cases of cutaneous lichen planus will spontaneously resolve within one year [2]. Therefore, treatment is directed at hastening the resolution of the disease. The first-line therapy for cutaneous lichen planus is high potency topical steroids. In cases that cannot be adequately controlled with topical steroids, other treatments such as oral glucocorticoids, phototherapy, and oral retinoids can be used as second-line therapy. Other medications that have been reported to have some benefit in treating cutaneous lichen planus include hydroxychloroquine, sulfasalazine, cyclosporine, and azathioprine [2]. Antihistamines have been shown to be useful for symptomatic treatment of pruritis. In patients with HCV, it is still unclear whether treatment of HCV infection leads to resolution of lichen planus. Some studies have found resolution of lichen planus with interferon treatment in HCV patients, while others have reported exacerbation of symptoms [8].

## Conclusions

Disseminated or eruptive lichen planus is a rare variant that has not been well studied, and little is known about the etiologies and treatments of this rare form. The presentation of eruptive lichen planus can be misleading at first as it often presents as a rapidly spreading exanthem, unlike classical lichen planus which presents more insidiously. It is imperative that family physicians consider lichen planus on the differential when a patient with known HCV infection presents with a rapidly spreading exanthem. Although screening patients diagnosed with lichen planus for HCV is controversial, it is something for family physicians to consider given that proper diagnosis of HCV infection can lead to earlier treatment and potentially better outcomes with chronic HCV infection.
